# Venezuelan Equine Encephalitis in Panama: Fatal Endemic Disease and Genetic Diversity of Etiologic Viral Strains

**DOI:** 10.1371/journal.pntd.0000472

**Published:** 2009-06-30

**Authors:** Evelia Quiroz, Patricia V. Aguilar, Julio Cisneros, Robert B. Tesh, Scott C. Weaver

**Affiliations:** 1 Gorgas Memorial Institute, Panama City, Panama; 2 Center for Tropical Diseases, University of Texas Medical Branch, Galveston, Texas, United States of America; 3 Departments of Microbiology and Immunology, University of Texas Medical Branch, Galveston, Texas, United States of America; 4 Pathology, University of Texas Medical Branch, Galveston, Texas, United States of America; Pediatric Dengue Vaccine Initiative, United States of America

## Abstract

Venezuelan equine encephalitis (VEE) is a reemerging, mosquito-borne viral disease of the neotropics that is severely debilitating and sometimes fatal to humans. Periodic epidemics mediated by equine amplification have been recognized since the 1920s, but interepidemic disease is rarely recognized. We report here clinical findings and genetic characterization of 42 cases of endemic VEE detected in Panama from 1961–2004. Recent clusters of cases occurred in Darien (eastern Panama) and Panama provinces (central Panama) near rainforest and swamp habitats. Patients ranged from 10 months to 48 years of age, and the more severe cases with neurological complications, including one fatal infection, were observed in children. The VEE virus strains isolated from these cases all belonged to an enzootic, subtype ID lineage known to circulate among sylvatic vectors and rodent reservoir hosts in Panama and Peru. These findings underscore endemic VEE as an important but usually neglected arboviral disease of Latin America.

## Author Summary

Venezuelan equine encephalitis (VEE) is a mosquito-borne viral disease that has caused major epidemics in many parts of Latin America and has even spread into Texas on one occasion. These epidemics result from spillover to humans of a horse-mosquito-horse amplification cycle that has occurred periodically since the 1920s. However, between these equine-mediated epidemics, little attempt is typically made to detect VEE in humans. Here, we show that VEE virus strains that typically circulate in a mosquito-rodent cycle, termed enzootic strains, also produce many cases of severe and sometimes fatal disease in Panama in the absence of apparent epidemics. These endemic infections are probably rarely detected because they are difficult to distinguish clinically from dengue fever, another mosquito-borne viral illness common in the tropics. Our findings underscore endemic VEE as an important but usually neglected arboviral disease of Latin America.

## Introduction

Venezuelan equine encephalitis virus (VEEV) is the most important alphaviral (*Togaviridae*: *Alphavirus*) pathogen of humans and domestic animals in the western hemisphere [1],[2]. Most human and animal disease occurs when VEEV undergoes an amplification cycle where equids (horses, donkeys and mules) become infected and develop high titer viremia, facilitating transmission by *Aedes* and *Psorophora* spp. mosquitoes to susceptible equids or people. The VEEV strains involved in this equid amplification cycle belong to subtypes IAB and IC, and are called epizootic or epidemic (henceforth called epidemic). These strains arise periodically and repeatedly via mutation of enzootic VEEV subtypes [3]. The resulting epidemics typically involve tens-to-hundreds of thousands of cases in humans and equids. Human disease is highly debilitating and immunosuppressive, and occasionally fatal (less than 1% of cases); however, many persons, especially children, suffer permanent neurological sequelae [2],[4]. In equids, VEEV causes mortality in roughly half of infected animals, resulting in major effects on agricultural activities that rely on these animals in many parts of Latin America.

The progenitors of the epidemic VEEV strains are called enzootic viruses, are not associated with equine disease, and circulate more or less continuously in forested or swamp habitats where rodents serve as the reservoir hosts and mosquitoes in the subgenus *Culex* (*Melanoconion*) act as vectors [5]. These enzootic strains were first isolated in the 1950's, and a fatal human isolate made in Panama in 1961 [6] was later identified as a subtype ID enzootic strain. The enzootic VEEV cycle, which involves subtypes ID and IE, often goes undetected because these viruses are incapable of exploiting these hosts for amplification to produce widespread disease [2],[4]. However, enzootic VEEV strains are increasingly recognized as important endemic pathogens of people who live near the enzootic transmission foci and/or enter the habitats where enzootic circulation occurs [4],[7],[8]. Because human VEE is nearly impossible to distinguish clinically from other tropical viral diseases such as dengue, most cases are probably undiagnosed. Endemic VEE is therefore grossly underreported in many parts of the Americas where enzootic circulation occurs and surveillance for febrile illness is limited.

To further characterize human disease caused by enzootic VEEV, we studied 42 cases of VEEV infection characterized by virus isolation, some accompanied by neurological disease, detected in Panama from 1961–2004. Here we describe the clinical signs and symptoms of disease, ecological conditions accompanying VEEV exposure and genetic characterizations of the etiologic VEEV strains.

## Methods

### Virus isolates

VEEV isolates from infected persons, included in the study, are shown in Table 1. The retrospective analyses of these isolates and reporting of clinical data were approved by the Institutional Review Boards of the Gorgas Memorial Institute and the University of Texas Medical Branch. Most of the viruses were isolated in Vero cells or by intracerebral inoculation of newborn mouse brains with sera from febrile patients, with triturated mosquito pools, or with rodent tissues (approved by the institutional animal care and use committees of the Gorgas Memorial Institute and the University of Texas Medical Branch). Supernatant fluids from infected cells or brain homogenates were stored at −70°C and later used for the extraction of viral RNA.

**Table 1:** Venezuelan equine encephalitis virus isolates from humans, mosquitoes and rodents included in the study.

Strain	GenBank Acc. No.*	Location (Province, site)	Year of isolation	Host	Age (Years)**	Passage history	Signs and symptoms***
4840	FJ969477	Panama	1961	Human	unknown	BHK-1, SMB-2,V-1, CEC-1	unknown
BT-2607	AF004450	Bocas del Toro, Almirante	1961	Mosquito	NA	?, BHK-1	NA
3880	L00930*	Panama, Cañito	1961	Human	14	SMB-2, V-2	1, 2, 4, 5, 10, 19***
8137	U88634*	El Rincón	1962	Human	unknown	SMB-1, V-1	unknown
8138	FJ969478	El Rincón	1962	Human	unknown	CEC-2	unknown
8585	FJ969479	El Rincón	1962	Human	unknown	Unknown	unknown
202330	U88635*	Panama, Gamboa	1963	Rodent	NA	SMB-1, V-2	NA
P. Quintero	U88636*	Panama, Juan Mina	1964	Human	unknown	CEC-1, V-1	unknown
F-322	U88638*	Panama, Cañito	1965	N.K.	unknown	Unknown	unknown
240832	U88637*	Panama, Gamboa	1965	N.K.	unknown	CEC-1, V-1	unknown
MARU23283	FJ969485	Panama, Bayano	1977	Human	unknown	Unknown	unknown
GML903104	U88639*	Panama, Bayano	1977	Mosquito	NA	Unknown	NA
MARU29136	FJ969469	Panama, Alto de Jobo	1981	Human	unknown	Unknown	unknown
GML903837	FJ969476	Panama, Bayano	1984	Mosquito	unknown	Unknown	unknown
GML903843	U88640*	Panama, Bayano	1984	Mosquito	unknown	V-1, BHK-1	unknown
487317	FJ969467	Veraguas Santiago	1990	Human	47	V-3	1, 2
489243	FJ969474	Panama, San Miguelito Chilibre	1991	Human	>15	V-4	1, 2, 3, 4, 5, 6, 7, 8
93P1513	U88641*	Panama, Lake Gatun	1993	Human	27	SMB-1, V-1	unknown
489245	FJ969472	Panama, Felipillo	1995	Human	21	V-4	1, 2, 3
489246	FJ969470	Cocle, Aguadulce	1995	Human	24	V-4	1, 2, 9
489247	FJ969471	Panama, Felipillo	1996	Human	>15	V-4	1, 9, 10
474590	FJ969449	Panama, Mananitas	1997	Human	16	V-2	1, 2, 3, 9, 11, 12, 16
487315	FJ969463	Panama, Felipillo	1997	Human	28	V-3	1, 2, 3, 4, 5, 6, 9, 10, 11, 13
487308	FJ969457	Darien, Sambu	1998	Human	10	V-2	1, 2, 3, 4, 11
487309	FJ969458	Darien, Sambu	1998	Human	16	V-2	1, 2, 9, 11
487310	FJ969459	Darien, Sambu	1998	Human	15	V-3	1, 2, 9, 11, 13
487311	FJ969460	Darien, Sambu	1998	Human	9	V-3	1, 2, 11
487312	FJ969461	Darien, Sambu	1998	Human	23	V-3	1, 2, 4, 10
487313	FJ969462	Darien, Sambu	1998	Human	39	V-3	1, 9, 10, 11
487314	FJ969466	Darien, Sambu	1998	Human	18	V-3	1, 2, 10
481460	FJ969450	Panama, Felipillo	2000	Human	16	V-2	1
484551	FJ969451	Darien, R Iglesias	2001	Human	15	V-2	1
484918	FJ969452	Darien, Yaviza	2001	Human	5	V-2	1
485028	FJ969453	Darien, Yaviza	2001	Human	9	V-2	1
485029	FJ969454	Darien, Yaviza	2001	Human	9	V-2	1
485030	FJ969455	Darien, Yaviza	2001	Human	3	V-2	1
489242	FJ969475	Darien, R Iglesias	2001	Human	10	V-3	1
487321	FJ969465	Darien, Yaviza	2001	Human	5	V-3	1, 5, 15, 19
486729	FJ969456	Darien, Meteti	2002	Human	20	V-1	1, 2, 3, 4, 5, 6
212857	FJ969484	Darien, Santa Fe	2003	Human	10	SMB-1	1, 2, 3, 5, 14
212863	FJ969480	Darien, Santa Fe	2003	Human	2	SMB-1	1, 5
212908	FJ969482	Darien, Santa Fe	2003	Human	15	SMB-1	1, 2, 4, 11, 14, 17
213391	FJ969483	Bocas del Toro, Guabito	2003	Human	13	SMB-1	16, 18
213413	FJ969481	Panama, Pacora	2003	Human	26	SMB-1	1, 2, 3, 10, 11
490021	FJ998042	Panama, El Cacao	2004	Human	12	unknown	16
490006	FJ998043	Panama, La Trinidad	2004	Human	12	unknown	1
489607	FJ998044	Colon, Escobal	2004	Human	48	unknown	1

*: Sequence determined previously.

**: NA = not applicable.

***: 1 = Fever; 2 = headache, 3 = tremors; 4 = nausea; 5 = vomiting; 6 = diarrhea; 7 = rash; 8 = renal/hepatic failure; 9 = arthralgia; 10 = myalgia; 11 = retroorbital pain; 12 = exanthema; 13 = sore throat; 14 = back pain; 15 = convulsion; 16 = encephalitis; 17 = cough; 18 = hemorrhage; 19 = fatal.

***: Data previously reported by Johnson et al. [6].

### Plaque assays

To determine the viremia levels in infected patients, available sera were tested by plaque assay as previously described [9] using Vero cells.

### Viral RNA extraction and cDNA synthesis

RNA was extracted from virus stocks as described previously [10]. A 250 µl volume of the 10% homogenized mouse brain tissue or supernatant was mixed with 750 µl of Trizol LS (Gibco-BRL, Gaithersburg, MD) and RNA was extracted following the manufacturer's protocol. Synthesis of cDNA was performed by mixing 5 µl of the RNA (1/10^th^ of the extracted RNA) with 1 µmol of reverse primer V9207B(−), 1× First Strand Buffer (Gibco), 1 mM dNTPs, 80 U RNAsin (Promega, Madison, WI) and 200 U SuperScript II Reverse Transcriptase (Gibco). The reaction was incubated at 42°C for 1 h.

### PCR amplification, sequencing and phylogenetic analysis

PCR was carried out using primers V8369(+) (GAGAACTGCGAGCAATGGTCA) and V9207B(−) (TRCACTGGCTGAACTGTT) as previously described [8]. These primers amplify the N-terminus of the PE2 envelope glycoprotein precursor gene, a region that has been used extensively in VEEV phylogenetic analyses [8],[11],[12] and is known to undergo critical amino acid substitutions associated with epidemic VEEV emergence [10]. The 816 bp PCR product was sequenced directly using primers V8659 (AATTGAGGCAGTGAAGAGCGAC) and V8953B (CTGCCTACAGGATTAAAT) using an Applied Biosystems (Foster City, CA) Prism automated DNA sequencing kit following the manufacturer's protocol. Sequences were aligned using MacVector program (Oxford Molecular Group, Campbell, CA) and phylogenetic analyses were performed using the maximum parsimony and neighbor joining programs implemented in the PAUP 4.0 software [13] or with MrBayes 3.0 [14] with 1,000,000 iterations. Bootstrap analyses were conducted using neighbor joining with 1,000 replicates to place confidence values on grouping within the trees [15].

## Results

### Distribution of the VEEV cases in Panama

**Figure 1:**
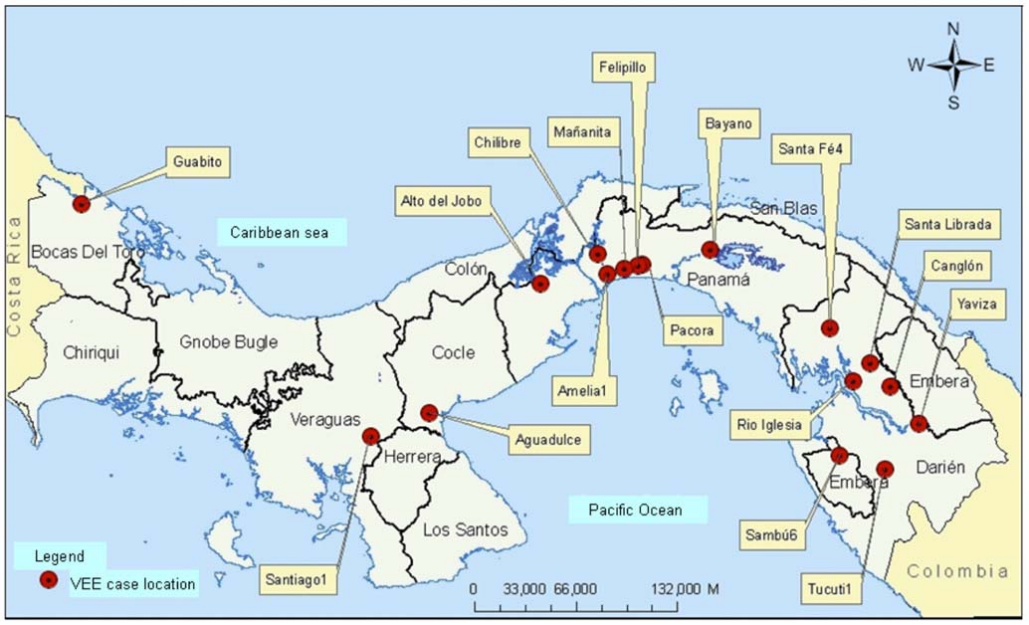
Map of Panama showing the locations of the Venezuelan equine encephalitis cases.

Data from recent VEE cases we studied (1990–2004) were compared with those from the literature dating to 1961. Recent VEE cases in Panama were reported in the provinces of Darien, Panama, Cocle, Veraguas and Bocas del Toro (Figure 1). Two clusters of cases were observed - one covering the Darien province and the second covering Panama Province (central Panama). Darien, the largest and most sparsely populated of the regions, extends from the hinterlands of Panama Province to the Colombian border, comprising more than one-third of the national territory. The land in Darien is characterized by forest and swamps and thus includes large areas of habitat for the *Culex* (*Melanoconion*) vectors of enzootic VEEV and small rodents that serve as the principal reservoir host [16]. The second cluster of cases was observed in Panama Province, between Gatun and Bayano Lakes. The surrounding area is characterized by rainforest and swamps habitats and is also conducive to enzootic VEEV circulation [6],[17],[18],[19]. Cases of VEE in Panama were reported in patients whose age ranged from 10 months to 48 years old. The more severe cases with neurological complications were observed in children.

### Description of the VEE cases

The VEE cases we studied, as well as those previously reported and included here for comparative purposes, were characterized by fever, headache, tremors, nausea, vomiting and diarrhea (Table 1). Only 3 of the cases developed neurological complications, and 2 of these were fatal. All cases of neurological disease were children less than 15 years of age. Overall, the most common signs and symptoms among the 33 VEE patients for which data were available included fever (94%), headache (55%), retroorbital pain (27%) and tremors (27%)(Table 2). These common signs and symptoms would not distinguish VEE from a wide variety of other tropical diseases, including malaria and dengue, without laboratory diagnostics. The overall case fatality rate of the Panamanian cases with known outcomes was 2/39, or 5% (Table 1). This rate is higher than that reported during most VEE epidemics (typically less than 1%) [20], which could reflect greater recognition of milder cases when more active surveillance is being conducted during outbreaks.

**Table 2:** Clinical signs and symptoms in 33 cases of Venezuelan equine encephalitis evaluated in Panama from 1961–2004.

Sign/symptom	% of patients exhibiting
Fever	94
Headache	55
Retroorbital pain	27
Tremors	27
Nausea	21
Vomiting	21
Arthralgia	21
Myalgia	21
Diarrhea	9
Encephalitis	9
Sore throat	6
Back pain	6
Rash	3
Renal/hepatic failure	3
Exanthema	3
Convulsions	3
Cough	3
Hemorrhage	3
Fatality	6

### Viremia titers

To investigate whether humans infected with subtype ID enzootic VEEV develop viremia titers sufficient to infect potential mosquito vectors, we quantified the virus titers in the sera of 5 patients (the only ones from which virus was isolated that were still available) using plaque assay. Titers varied from 3.0×10^2^ to 6.7×10^5^ PFU/ml (Table 3). Because the sera may not have been sampled at the time of peak viremia, these titers may underestimate maximum viremia levels.

**Table 3:** Viremia titers in febrile patients infected with VEEV.

Code	Serum titer (PFU/ml)
213413	2×10^2^
213388	<10^2^
212857	7×10^3^
212908	1.2×10^3^
212860	<10^2^
213391	6.7×10^5^
212863	2.0×10^4^

### Phylogenetic analyses

**Figure 2:**
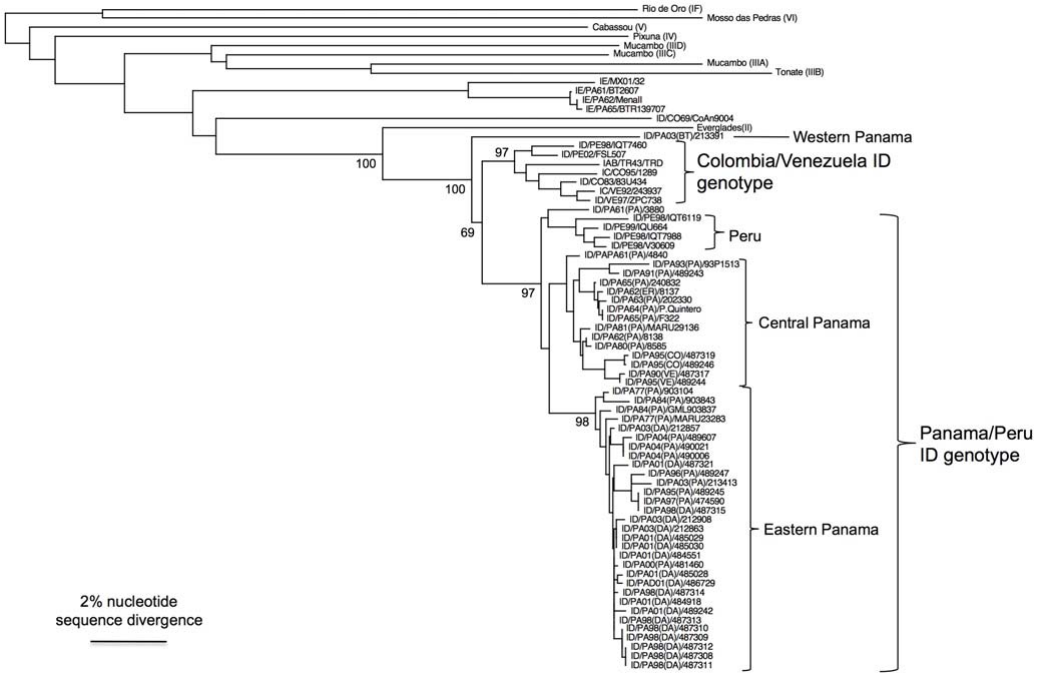
Phylogenetic tree generated from partial PE2 gene sequences of Panamanian VEEV strains and homologous VEE complex alphavirus sequences from the GenBank library. The tree was produced using the neighbor joining program implemented in PAUP 4.0 [13]. VEEV strains are denoted by abbreviated country and year of isolation, followed by strain designation. Panamanian strains include abbreviated province in parentheses as follows: BT – Bocas del Toro; COC – Cocle; COL – Colon; DA – Darien; PA – Panama; VE – Veraguas. Numbers indicate bootstrap values for clades.

To investigate genetic relationships among the VEEV strains from Panama in comparison to others isolated from different regions of the Americas, phylogenetic analyses were performed. Maximum parsimony, neighbor joining and Bayesian methods all generated similar tree topologies. The neighbor-joining tree based on the partial PE2 sequences (Figure 2) showed that most of the subtype ID VEEV strains from Panama formed a single clade, with previously sequenced Panamanian subtype ID strains as well as some Peruvian ID isolates. This group was previously called the Panama-Peru subtype ID genotype [21]. Within this group, 2 distinct clades among the Panamanian strains were observed: 1) strains from Central Panama; 2) isolates from Eastern Panama (Darien). The final subtype ID isolate, strain 213391 from western Panama (Bocas del Toro Province), was phylogenetically distinct from all other ID isolates. Our phylogenies placed it outside of the Panama/Peru genotype, but this placement was supported by a bootstrap value of only 69%. The location of collection of this strain was geographically close to those of subtype IE strains isolated in the 1960s (Fig. 2). Therefore, the geographic ranges of subtypes ID and IE may overlap slightly.

## Discussion

In 1961, the first evidence of VEEV in Panama was obtained when the virus was isolated from a fatal human case. The patient, a 14-year-old boy, resided in Cañito, near Gatun lake [6]. Since then, febrile disease associated with VEEV has been reported in the vicinity of Gatun and Bayano Lakes and in the province of Darien, all areas of rainforest and swamp habitat. Our results indicate that VEEV infection in at least 2 regions of Panama may be common, and the clinical features of the disease evaluated in 33 acute individuals suggest that only laboratory diagnostics can distinguish VEE from other febrile, tropical diseases such as malaria, dengue and Mayaro fever that present with overlapping signs and symptoms. Thus, the incidence of VEE in Panama and other endemic regions of Latin America is probably grossly underreported because malaria and dengue are assumed to be the etiologies of all febrile illnesses in the absence of laboratory diagnostics. Because exposure to VEEV involves vectors with different temporal and spatial distributions than those of dengue and malaria [20],[22], improved VEE diagnosis could lead to better prevention methods to reduce human exposure.

For subtype ID VEEV, the highest viremia titers that we measured in infected persons are presumably capable of orally infecting and being transmitted by several mosquito species including the proven subtype IE enzootic VEEV vector *Cx.* (*Mel.*) *taeniopus* [23], and the proven and potential epidemic VEEV vectors *Psorophora confinnis* [24], *Ae. taeniorhynchus* [25],[26], *Ae. albopictus* [27] and *Ae. aegypti* [28]. Although infection thresholds have not been determined for natural, enzootic subtype ID vectors, we believe that it is likely that they could be infected by feeding on persons with titers on the order that we measured. Furthermore, serum titers and mosquito transmission potential could be even higher if the individuals we studied were not sampled at the peak of viremia. Previous studies have reported that horses infected with epidemic VEEV strains develop comparable viremia levels, which allow them to serve as highly efficient amplifying hosts by infecting susceptible mosquitoes [29],[30],[31]. These results suggest the possibility that humans infected with subtype ID VEEV in Panama could support an epidemic human-mosquito-human transmission cycle, especially if a viremic individual reached an urban area with abundant *Ae. aegypti* or *Ae. albopictus*. These species, especially the former, are highly efficient vectors of other human arboviruses such as dengue [32] and chikungunya [33] due to their ecology and behavior as well as their susceptibility to infection. Interestingly, VEE is endemic in Iquitos, Peru, where *Ae. aegypti* is abundant and transmits DENV to thousands of people during epidemics. However, the average number of diagnosed VEE human cases per year in Peru is about ten, but this is probably a gross underestimate of the true number (P.V. Aguilar, unpublished). Because VEEV viremia in humans is high enough to infect *Ae. aegypti*, regular human VEE outbreaks in Iquitos might be expected. However, only one VEE human outbreak has been reported in Iquitos (in 2006) [8] despite detection of endemic VEE there since 1993 [12]. Thus, additional factors not yet understood may be crucial to the emergence of enzootic VEE. Mutations that arise during the adaptation of the virus to certain mosquito species have been previously described as a key factor in the epizootic VEE emergence, and thus deserve further study [1],[34]. Other factors such as mosquito abundance should also be investigated.

Hemorrhagic manifestations among VEE cases are rare. To date, only two patients (<15 years of age) have been reported with hemorrhagic complications upon VEEV infection with the subtype ID Panama/Peru genotype [35]. Interestingly, one of the VEE patients from Panama that we studied also reported hemorrhagic manifestations and encephalitis, although the former was not determined to be the cause of death. Thus, although hemorrhages associated with VEEV infection are unusual, they do occur and their potential role in a fatal outcome should be investigated.

One of the Panamanian patients infected with VEEV from 1990–2004 developed a fatal encephalitic disease, further confirming that subtype ID Panama/Peru genotype can cause fatal disease. Although subtype ID Panama/Peru lineage strains have been continuously associated with human illness in Peru, fatal disease has never been reported there despite many cases in children [8]. The reasons for this lack of fatal VEE in Peru remain unknown, but could include underreporting in many locations where laboratory diagnosis is not readily available.

Our phylogenetic analyses revealed that all Panamanian isolates with one exception belong to the previously described Panama/Peru genotype [21],[36]. Within this group, 2 distinct clades among the Panamanian strains were observed: one sampled from Central Panama, and a second from Eastern Panama (Darien). A third Panamanian ID lineage was identified in western Panama (Bocas del Toro), which may represent a new, major lineage in subtype ID. Longer sequences may be needed to definitively determine the relationship of this new lineage to the other ID genotypes. Interestingly, this Bocas del Toro isolate was recovered from a patient who presented with encephalitis and hemorrhagic complications. Thus, further studies are needed to investigate whether this specific genotype is particularly associated with severe encephalitis and hemorrhagic disease. In addition, future genetic analyses of the Panamanian and Peruvian isolates associated with hemorrhagic disease could provide important insights about the viral determinants (if any) of this infrequent disease manifestation. Potential host factors/conditions should also be investigated for their role in disease outcome.

## Supporting Information

Alternative Language Abstract S1 Translation of abstract into Spanish by Patricia Aguilar
(0.03 MB DOC)
