# Supplementary material for: Venezuelan Equine Encephalitis in Panama: Fatal Endemic Disease and Genetic Diversity of Etiologic Viral Strains
Source: PLoS Negl Trop Dis. 2009 Jun 30;3(6):e472. doi: 10.1371/journal.pntd.0000472 (PMC2697379; doi:10.1371/journal.pntd.0000472)
Supplement: Alternative Language Abstract S1 — Translation of abstract into Spanish by Patricia Aguilar (0.03 MB DOC) [file pntd.0000472.s001.doc]

Abstract in Spanish

La encefalitis equina venezolana (EEV) es una enfermedad re-emergente en las Americas que es transmitida por artrópodos. Esta enfermedad es sumamente debilitante y en ocasiones fatal para los humanos. Las epidemias periódicas mediadas por la amplificación en equinos han sido reconocidas desde 1920, pero los casos que se presentan entre epidemias son raramente reconocidos. El presente trabajo describe la sintomatología clínica y la caracterización genética de 42 casos de EEV endémico detectados en Panamá entre los años 1961 al 2004. Focos recientes de casos ocurrieron en Darien (lado este de Panamá) y las provincias de Panamá (zona central de Panamá) cerca a bosques y pantanos. La edad de los pacientes fluctuó entre los 10 meses y los 48 años, y los casos más severos con complicaciones neurológicas, incluyendo una infección fatal, fueron observados en niños. Los virus de EEV aislados de estos pacientes fueron identificados como el subtipo enzootico ID, que se conoce circula entre los vectores selváticos y los roedores que son los huéspedes reservorios en Perú y Panamá. Los resultados destacan el EEV endémico como una enfermedad arboviral importante, pero frecuentemente desatendida en Latinoamérica.
